# Integrating active and passive environmental DNA sampling into coral reef monitoring: implications for community-based stewardship in Hawai i

**DOI:** 10.21203/rs.3.rs-9580292/v1

**Published:** 2026-06-19

**Authors:** Cécile M Vimond, Zoe Hill, Cameron AJ Walsh, Pamela Weiant, Kēhaulani Kupihea, Peter B Marko, Patrick K Nichols

**Affiliations:** University of Hawai i at Mānoa; University of Hawai i at Mānoa; University of Hawai i at Mānoa; Mālama Maunalua; Mauliola Ke ehi; University of Oregon; University of Oulu

**Keywords:** Molecular biomonitoring, multimarker, local ecological knowledge, ecosystem stewardship, coral reefs, community composition

## Abstract

**Background:**

Environmental DNA (eDNA) has transformed biodiversity monitoring, but its application for long-term ecological assessment remains limited by uncertainty in the temporal stability and consistency of eDNA signals, as well as by specialized workflows that limit accessibility for community-led programs. Expanding participation in biomonitoring requires approaches that are both logistically feasible and scientifically robust. Here, we evaluated eDNA tools for coral reef monitoring in Hawai i by comparing (1) coral-specific genetic markers for tracking benthic community dynamics through time, and (2) passive eDNA samplers (PEDS) as a low-effort alternative to active filtration. This research was conducted with local stewardship organizations on O ahu, Hawai i, positioning eDNA as a complementary tool to place-based ecological knowledge that can inform conservation strategies and enhance ecological monitoring.

**Results:**

Across five broad taxonomic markers, we detected diverse reef communities spanning 17 phyla, with consistent regional differences in community composition, including clear differentiation in algal assemblages. Passive samplers recovered comparable taxonomic richness to active filtration when sampling effort was standardized, but showed greater variability among replicates, whereas active methods more consistently detected rare and transient taxa. Despite this, passive samplers captured similar broad-scale community patterns with reduced logistical effort. For coral monitoring, all three markers (12S, 16S, ITS2) showed strong correlations with visual cover and detected more genera than visual surveys. Among these, 16S showed the strongest and most consistent performance across temporal replicates, while ITS2 showed greater temporal variability.

**Conclusions:**

Accessible eDNA approaches can generate ecologically meaningful data for reef monitoring when matched to specific objectives. Passive samplers provide a scalable, low-barrier option for widespread community biomonitoring, while active filtration remains important for detecting rare taxa. A multimarker approach enabled detection of native, endemic, and nuisance taxa, offering an ecosystem-level perspective for management. Given the relatively slow pace of change in coral assemblages, periodic eDNA surveys may provide a useful and practical longer-term complement to rapid visual assessments of coral cover. Integrating these tools with local stewardship efforts can expand participation in eDNA biomonitoring while supporting conservation in urbanized, culturally significant reef systems.

## Background

Monitoring the composition and dynamics of ecological communities is fundamental to understanding ecosystem health and informing conservation planning [1–4]. On coral reefs, changes in community structure can signal shifts in ecosystem function, resilience, long before dramatic declines in coral cover become visually apparent [5–7]. However, many coral reef monitoring programs rely on visual surveys that, while effective for conspicuous taxa, can be time-intensive, selective across taxa, and limited in their ability to detect transient, rare, or early life-stage taxa [8–12].

Environmental DNA (eDNA) metabarcoding has emerged as a powerful complementary tool for ecological monitoring [13, 14], offering a non-invasive means of surveying biological communities from small volumes of water. Because organisms continually shed genetic material into their surroundings, eDNA integrates biological signals across time and microhabitats that are often inaccessible to visual census methods. On coral reefs, eDNA has been used to detect reef-building corals [15–17], cryptic invertebrates [18, 19], macroalgae [20], and fishes [21, 22], often capturing a broader snapshot of biodiversity than visual surveys alone [17, 23, 24]. Despite these advantages, many eDNA workflows remain technically and logistically demanding, limiting their adoption in community-led and resource-constrained monitoring programs.

For eDNA to be more broadly integrated into reef monitoring, surveys must be simple, cost-efficient, and standardized. Reef managers rely on repeated surveys to distinguish directional change from natural variability, particularly when evaluating coral decline, recovery, or the outcomes of restoration efforts [25–27]. Yet the stability of eDNA-based estimates of coral assemblages across time, seasons, and environmental conditions remains incompletely understood [28], especially on tropical fringing reefs where water movement, spawning events, and environmental forcing may influence eDNA production and persistence [29, 30].

An additional challenge lies in PCR metabarcoding primer selection (short DNA sequences that determine which taxa get amplified, hereafter referred to as “markers”). While universal markers enable broad community surveys [31], taxon-specific markers offer the potential to more precisely track focal groups such as reef-building corals [15–17]. Multiple coral-specific markers have been developed to target both mitochondrial and nuclear genes [15, 16, 32], yet their relative sensitivity, taxonomic resolution, and consistency through time vary among systems. Understanding how different coral markers perform under real monitoring conditions is therefore essential for translating eDNA data into management-relevant metrics such as coral richness, assemblage composition, and temporal change.

At larger spatial scales, reef management also requires baseline assessments of biodiversity across entire reef complexes. Multi-marker eDNA metabarcoding offers a broader perspective on reef ecosystems [31, 33], capturing taxa from microbes to megafauna that respond differently to stressors such as eutrophication and habitat degradation [34]. However, eDNA signals are inherently context-dependent, with patterns of detection influenced not only by methodological choices but also by site selection and temporal variability, including seasonal dynamics, hydrodynamic conditions, and episodic biological events such as spawning [30, 35–37]. As a result, surveys conducted at different times of year or across different reef habitats may recover distinct community signatures, even under otherwise comparable protocols.

To address the logistical challenges of repeated and spatially extensive sampling, there is growing interest in simplifying and automating eDNA workflows [38–42]. However, such simplification often involves trade-offs in how environmental DNA is captured and concentrated, which can influence detection probabilities and the ecological signal recovered from samples [43–46]. In particular, practical decisions regarding sampling design, including the choice between active filtration and passive DNA collection, can strongly influence both data quality and collection feasibility, particularly for long-term programs and community-led monitoring initiatives.

To address these complementary needs, this study was conducted in partnership with two community-based stewardship organizations in Hawai i (Mālama Maunalua and Mauliola Ke ehi) to evaluate eDNA approaches for coral reef monitoring. In Maunalua Bay, ongoing restoration efforts, such as invasive algae removal, outplanting of coral fragments, and recent designation as a Fisheries Management Area, necessitate baseline biodiversity surveys for tracking ecosystem-wide recovery. In Ke ehi, a culturally significant but heavily modified nearshore system [47–49], community partners seek accessible tools to document biodiversity and advocate for reef protection and restoration. Within this context, we evaluated whether eDNA approaches can support reliable and accessible reef monitoring in partnership with community stewardship organizations in Hawai i. Specifically, we (1) assessed the temporal performance of coral-specific markers for tracking coral assemblages, and (2) compared active filtration and passive sampling approaches for multi-taxa biodiversity surveys across reef systems. This framework provides practical guidance for integrating eDNA into coral reef monitoring and conservation efforts.

## Methods

### Coral time series - active filtration

**Figure 1:**
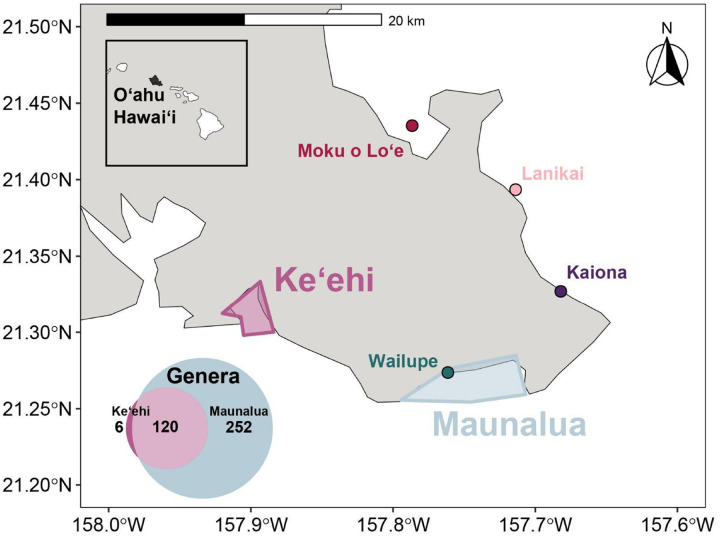
Map of eDNA sampling locations on O ahu, Hawai i. Four fringing reef sites (Moku o Lo e, Lanikai, Kaiona, Wailupe) were sampled seasonally (fall, winter, spring, summer) from 2019–2021 for temporal coral monitoring using coral-specific eDNA assays; visual assessments of coral cover were conducted at the initial and final sampling timepoints at the same four sites. Two embayments (Ke ehi and Maunalua) were subsequently surveyed once in 2025 for reef biodiversity using five metabarcoding markers, with parallel active (2-L filtration) and passive sampling approaches. Inset: Reef-associated genera detected by metabarcoding that were unique to, or shared between, the two regions.

Environmental DNA was repeatedly sampled from four fringing coral reef sites around O ahu, Hawai i (Fig. 1). Seawater samples were collected each season for two years (fall: Sept. 2019 & 2020; winter: Feb. 2019 & Jan. 2020; spring: May 2020 & April 2021; summer: Jun. 2020 & 2021). Shallow reef sites were selected to maximize variation in coral cover and community composition [17]. At each site, three replicate seawater samples were collected using 4-L collapsible containers (Hedwin Co., Baltimore, MD, USA) at ~ 1 m above the reef surface, with replicate containers spaced across the reef within 3 m of each other. Immediately following collection, the samples and a negative field blank control (see “Contamination prevention”, below) were placed in the dark on ice and filtered within 3 hr.

Water samples and field blanks were individually homogenized and a 2-L subsample was filtered through mixed cellulose ester filters (MilliporeSigma, Burlington, MA, USA; diameter 47 mm, pore size 0.22 μM) using a peristaltic pump (Cole-Parmer, Vernon Hills, IL, USA). Filters were then cut in half and placed into 2 mL screw-cap microcentrifuge tubes with 790 μL ATL lysis buffer (Qiagen, Hilden, DE) and 10 μL Proteinase K (Qiagen). Tubes were shaken vigorously for 5 min in a tissue lyser (Retsch, Haan, DE) and then incubated at 56°C for 30 min, followed by an additional 5 min in the lyser and 30 min incubation step. Samples were then frozen at −20°C for no longer than 24 h before proceeding with DNA extraction. Each filter half (technical replicate) per water sample (biological replicate) was separately extracted using Qiagen DNeasy Blood & Tissue kits by transferring 600 μL of the supernatant to new tubes and following the manufacturer’s protocol with minor adjustments: 600 μL AL Buffer, 600 μL ethanol, and two sequential elutions of 50 μL AE Buffer each for a final volume of 100 uL.

### Visual coral cover

Benthic community composition was assessed at the same four sites using high-resolution photographic quadrats along standardized underwater transects, once at the beginning (Sept. 2019) and once at the end of the coral eDNA sampling (June 2021). Three 30-m transect tapes were deployed perpendicular to shore, from back reef to fore reef, approximately 1–3 m depth. Along each transect, 0.25 m^2^ quadrat photographs were taken every 2 m using a Sony a7RII camera housed in an underwater housing. Images were uploaded to CoralNet [50–54] for annotation, and 100 random points per quadrat. Percent cover per genus was calculated from point annotations, excluding non-biological features such as tape, quadrat, or shadows.

### Community metabarcoding - active vs. passive eDNA filtration

Within two additional regions (Ke ehi: 7 sites; Maunalua: 12 sites), eDNA was collected once in 2025 using both active (Ke‘ehi: n = 14; Maunalua: n = 24) and passive (Ke‘ehi: n = 10, Maunalua: n = 24) filtration methods to compare community composition. However, at two sites in Ke ehi, active filtration was not logistically possible and were therefore sampled with passive samplers only. For active filtration, two seawater samples were collected and filtered as described above, using a portable peristaltic pump with dual pump heads (Proactive Environmental Products, Bradenton, FL, USA). At the same time, duplicate cotton membranes secured in passive environmental DNA filters (PEDS) were deployed at each site, suspended in the water column 1 m above the reef. After 30 min, PEDS were removed from the water and stored on ice in single-use, sealable plastic bags for no longer than 6 h before being stored at −20°C until laboratory processing. Active MCE filters were processed as described above in “*Coral time series active filtration*”. DNA was extracted from cotton membranes following the protocol for stationary PEDS [41], using a one cm strip (~1.5 g wet weight) cut from the center of thawed passive membranes and Qiagen Blood & Tissue kits.

### Metabarcoding and sequencing of libraries

DNA extracts from active and passive filter membrane replicates were then amplified using coral-specific as well as universal genetic markers for reef metazoans and macroalgae (Table 1). Triplicate amplifications were conducted in 13 μL volumes consisting of 6.3 μL MyTaq 2x (Bioline), 0.3 μL of each forward and reverse primers with built-in Illumina overhang adapters (10 μM), 0.65 μL bovine serum albumin (BSA, 20 mg/mL, Thermofisher Scientific), 4.45 μL nuclease-free water (Growcells), and 1 μL template DNA. Thermal cycling parameters consisted of profiles as reported by the assay authors [17, 55–58].

Triplicate PCR reactions were pooled by marker and the quality of pooled amplicons was verified with gel electrophoresis. All samples producing amplicons at the expected lengths were then prepared for sequencing by the Microbial Genomics and Analytical Laboratory at the University of Hawai i at Mānoa. Amplicons were purified using magnetic beads (1:1.2 DNA:bead concentration), indexed with the Nextera XT v2 (Illumina) adapters, and purified with an additional bead cleaning step. Amplicon DNA concentration was quantified using a Qubit fluorometer and dsDNA high-sensitivity detection kit (Thermofisher Scientific) and pooled to equimolar concentrations. Pooled libraries (including negative controls) were paired-end sequenced on an Illumina MiSeq platform using the V3 600-cycle reagent kit and ~ 12% PhiX spike at the Advanced Studies in Genomic, Proteomics, and Bioinformatics facility at the University of Hawai i at Mānoa.

### Contamination prevention

Filtration was performed in a lab physically isolated from all other molecular work. DNA extraction was conducted in site-specific batches by sampling date. All lab surfaces were bleach-decontaminated before and after filtering samples from a new site. Filtration equipment was flushed with 10% bleach for at least five minutes, followed by flushing with ~ 500 mL sample water prior to filtration. Field collection blanks consisted of an unused PEDS in a sealed plastic bag and bleach-decontaminated sample containers filled with 2-L tap water that were taken into and returned from the field (one per site per day). Tap water blanks were filtered through decontaminated equipment prior to biological samples. Unused PEDS were rehydrated with nuclease-free water and processed alongside biological replicates. Field blanks therefore served as a negative control for the eDNA collection and extraction steps. PCR negatives and blanks were processed alongside samples as a precaution to monitor potential cross-contamination and reagent carry-over. After use, all containers, filtering equipment, and tools such as forceps and scissors were decontaminated in a 10% bleach bath for 24 hr and air-dried. Containers used to collect field samples were randomly shuffled after cleaning to be used as collection blanks.

### Bioinformatics

Raw sequence datasets were interpreted using rainbow_bridge v1.33.8 [59], an optimized and enhanced version of the eDNAFlow pipeline [60] that uses Nextflow DSL2 [61]. All processing steps and parameter values described below were implemented within this workflow using default settings unless otherwise noted.. This workflow begins with quality filtering and paired-end merging using AdapterRemoval [62], followed by primer mismatch and minimum length filtering through OBITools [63]. Dereplication, clustering [64], and chimera filtering [65] using VSEARCH [66] yields zero-radius operational taxonomic units (zOTUs) with a minimum of eight reads across the library. A BLAST search [67, 68] through the “core_nt” database sourced from GenBank [69] is conducted for each zOTU where only hits with 100% query coverage are retained. Any zOTU with only one search hit at or above 97% identity is assigned to the matched taxon. When multiple taxa match at 97% similarity or above, the zOTU is collapsed to the lowest common taxonomic group of all matches within 1% identity of the top match [60].

The *insect* R package [70] was also used within the workflow to predict taxonomic identities for zOTUs with no BLAST hits of at least 97% similarity for markers with publicly available informatic sequence classification trees. To ensure that subsequent analyses focused on marine metazoans and macroalgae, zOTUs assigned to non-target taxa (e.g., bacteria, Aves, Insecta) or unassigned at the phylum level were manually removed from downstream analyses of community composition. For the coral-specific datasets, all zOTUs matching to Scleractinia were verified through a subsequent BLAST search. For each coral zOTU, we retained only the top BLAST hit (lowest e-value) genus present in our study region [71]. Contaminated reads that were identified in blank samples were removed using the decon function in the *microDecon* R package [72], with a zero-prevalence threshold of 0.7 and a relative abundance threshold of 0.5%.

### Statistical analysis

Statistical analyses were conducted independently on each marker in R v4.5.0 [73]. Generalized linear models (GLMs) were used to assess the relationship between percent visual coral cover and coral eDNA reads. Temporal consistency of eDNA detection was evaluated using two complementary approaches. First, temporal variability in abundance-based signal intensity was quantified using the coefficient of variation (CV) calculated for each genus × site × marker combination. To compare variability among markers, log-transformed CV values were analysed using linear mixed-effects models (LMMs; *lme4* package) [74], with marker identity as a fixed effect and site and genus included as random effects. Second, detection probability was modeled using a binomial generalized mixed-effects model (GLMM) with marker identity as a fixed effect and genus nested within site included as a random effect to account for spatial structure and non-independence of repeated measures across timepoints. Treating site as a fixed effect did not qualitatively change the results, indicating that inference was robust to this modeling choice.

Community diversity was quantified using Hill numbers estimated from read abundance data using *iNEXT* [75], including species richness (q = 0), Shannon diversity (q = 1), and Simpson diversity (q = 2), which differentially weight rare and common taxa. To ensure comparability among samples, diversity estimates were standardized to the minimum shared sample coverage using coverage-based rarefaction [76, 77].

For downstream compositional analyses, read counts from each marker were separately transformed using the eDNA index, a two-step process where reads are first normalized within samples and then rescaled per zOTU across all samples to adjust for differences in read depth and amplification efficiency [78]. The eDNA index was used to track within-taxon temporal changes in community composition, and no inferences were made regarding relative abundance among taxa.

Distance-based analyses were performed separately for each marker using Bray-Curtis pairwise dissimilarities on eDNA index-transformed read counts using the *vegan* R package [79, 80]. Permutational analysis of multivariate dispersion (PERMDISP) and permutational analysis of variance (PERMANOVA; 9999 permutations) were used to test the effect of region and filtration method on community dissimilarity. Community patterns were visualized with nonmetric multidimensional scaling (NMDS; k = 3, 1000 iterations).

Zeta diversity (ζ) was used to quantify shared diversity across multiple samples, capturing patterns of species retention and turnover [81]. Zeta decline across increasing orders (ζ_n_) was modeled separately for each marker using zeta.decline.ex in the *zetadiv* R package [82], with exponential and power-law models compared via the Akaike Information Criterion (AIC) to identify the best fit. These two functional forms represent distinct ecological processes of incidence-based turnover, with exponential decay reflecting more stochastic patterns of species loss across sites and power-law decay reflecting more structured turnover consistent with structured ecological and detection processes [83, 84].

## Results

### Coral cover and diversity

**Figure 2:**
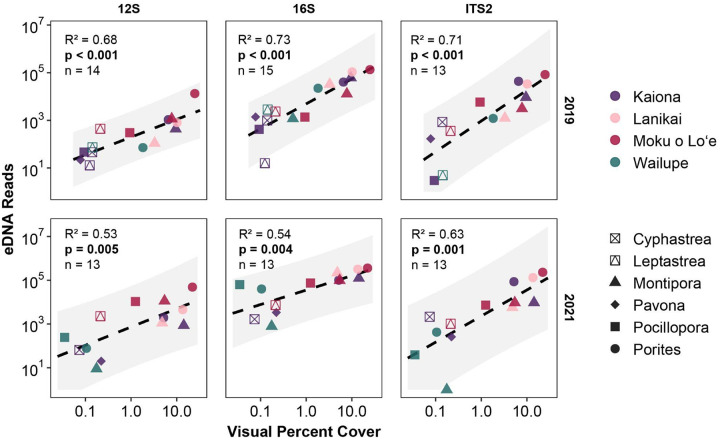
Correlation between visual percent cover and total eDNA reads across four sites on O ahu for coral-specific metabarcoding markers (12S, 16S, ITS2) from surveys conducted in 2019 and 2021. Points represent individual genera, colored by site with dashed lines showing the fitted linear regression (log-transformed), with gray shaded regions representing 95% prediction intervals.

Coral cover from visual surveys at four sites (Moku o Lo e, Lanikai, Kaiona, and Wailupe) was significantly correlated with eDNA reads for all three metabarcoding markers (GLM; pseudo-R^2^ = 0.53–0.73, p < 0.01; Fig. 2). The coral-specific markers recovered coral genera that were detected in visual surveys, with 12S detecting the greatest number of genera across sites and timepoints (**Table S1**). At the site level, eDNA consistently detected more genera than visual surveys (**Figure S1a**).

Variability differed among markers depending on the metric used, with less-abundant genera having patchy eDNA detections through time (**Figure S1b**). ITS2 had a greater coefficient of variation (CV) compared to 12S (**Figure S1c**), indicating greater proportional fluctuation in signal intensity, while 16S did not differ significantly from 12S (LMM; ITS2: p < 0.001; 16S: p = 0.30; **Table S2**). Temporal detection frequencies also differed significantly among markers, with 16S having greater detection probabilities than 12S (p < 0.001), while ITS2 exhibited significantly lower detection probabilities than 12S (p < 0.001; **Table S2**).

#### Reef flora and fauna communities

**Figure 3:**
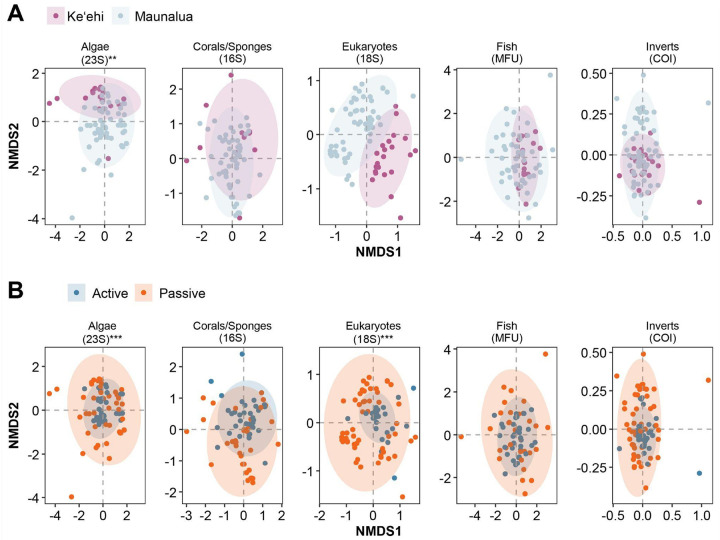
Non-metric multidimensional scaling of community dissimilarity based on (a) survey region (Ke ehi and Maunalua Bay) and (b) eDNA sampling methodology (active vs. passive filtration) for ordinations of each of the five genetic markers tested in 2025: algae (23S), corals/sponges (16S), total eukaryotes (18S), fish (MFU), and macroinvertebrates (COI). Two-dimensional stress is listed by marker and asterisks indicate the degree of significance for community dissimilarity: p < 0.001***; p < 0.01**.

Metabarcoding of eDNA from five broad-scale markers detected 17 metazoan and macroalgal phyla across all samples at Ke ehi and Maunalua from 2025. Significant community dissimilarity was observed between regions (Ke ehi and Maunalua) for the algal (23S) marker (PERMANOVA; 23S: R^2^ = 0.04, pseudo-F = 3.29, p = 0.006; Fig. 3a) and between active and passive filtration methods for the eukaryote (18S) and algal (23S) markers (PERMANOVA; 18S: R^2^ = 0.02, pseudo-F = 3.09, p < 0.001; 23S: R^2^ = 0.02, pseudo-F = 1.73, p < 0.001; Fig. 3b). Tests for homogeneity of dispersion were not significant among groups (PERMDISP; p > 0.05).

**Figure 4:**
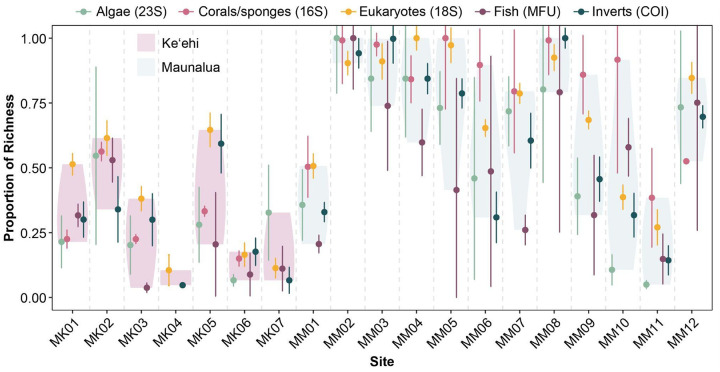
Violin plots of coverage-standardized sample zero-radius operational taxonomic unit (zOTU) richness (q = 0) across sites from within regions (Ke ehi: MK; Maunalua: MM). Points representing the mean and standard error of samples within each genetic marker: algae (23S), corals/sponges (16S), total eukaryotes (18S), fish (MFU), and macroinvertebrates (COI).

Observed taxonomic richness varied considerably across sites and markers. Greater richness was recovered in Maunalua than in Ke ehi, with one site (MM02) exhibiting consistently high richness across all markers (Fig. 4). Maunalua also harbored many more unique genera than Ke ehi (252 vs. 6; Fig. 1). Active filtration of eDNA detected a greater number of unique taxa than the passive method (**Figure S2**, **Table S3**).

**Figure 5:**
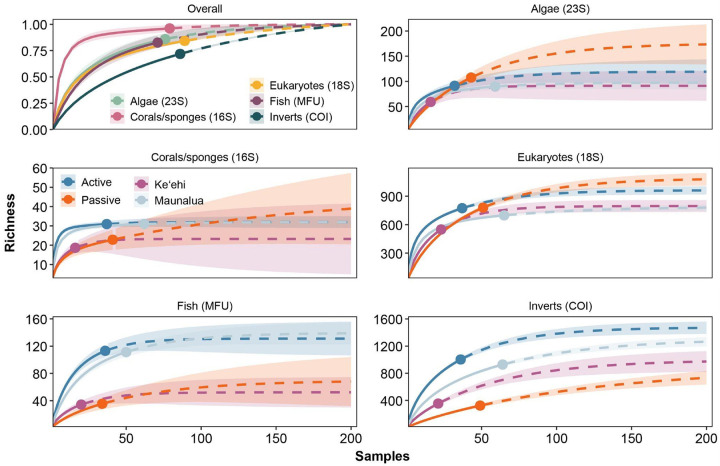
Observed and extrapolated estimates of zero-radius operational taxonomic unit (zOTU) richness between regions (Ke ehi and Maunalua) and environmental DNA filtration methods (active vs. passive, pooled between regions) for each genetic marker: algae (23S), corals/sponges (16S), total eukaryotes (18S), fish (MFU), and macroinvertebrates (COI).

Because sampling effort varied across regions and filtration methods, we used coverage-based standardization to enable direct comparison of alpha diversity. When standardized to the minimum shared sample coverage (i.e., holding sampling completeness constant across comparisons), Maunalua had greater richness than Ke ehi for most markers (Fig. 4). However, extrapolated asymptotic richness estimates (i.e., projected richness at 100% coverage) revealed a different pattern: the two regions had comparable extrapolated taxonomic richness for most markers except for fishes (MFU) and macroinvertebrates (COI) (Fig. 5, **Table S4**). Similarly, while active filtration detected more unique taxa in observed richness counts, extrapolations show that passive sampling could recover greater richness, again excepting taxa detected using MFU and COI markers (Fig. 5, **Table S4**).

**Figure 6:**
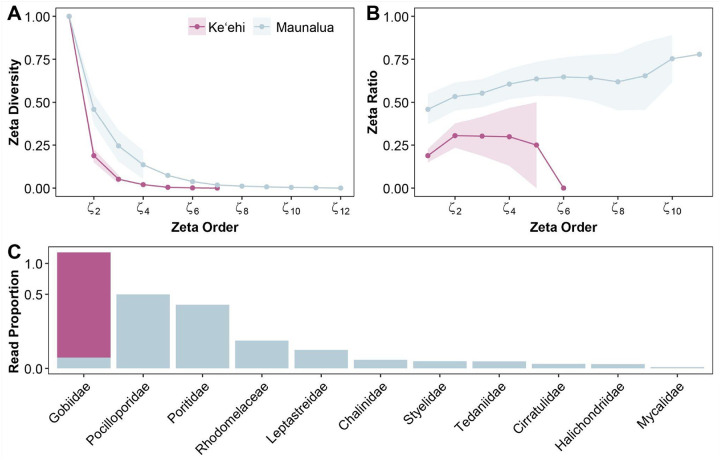
Zeta diversity across sample sites and core taxa by embayment region (Maunalua vs. Ke‘ehi). (a) Decline in shared zero-radius operational taxonomic units (zOTUs) and (b) retention ratio across zeta orders. (c) Relative eDNA read proportions for core taxa by sampling region (Ke ehi and Maunalua), identified to family. Bars indicate contributions from within each region.

Maunalua exhibited greater zeta diversity (Fig. 6a), indicating that more taxa were consistently detected across sites within each region. Core taxa in Maunalua (i.e., those detected at every site within the region, summarized at the family level to account for variation in taxonomic resolution and assignment confidence across markers) included reef-building corals (Pocilloporidae, Poritidae, Leptastreidae), filter feeders (Chalinidae, Halichondriidae, Mycalidae, Styelidae), deposit feeders (Cirratulidae), bioeroders (Tedaniidae), macroalgae (Florideophyceae), and cryptic microcarnivores (Gobiidae). In contrast, Ke ehi exhibited a steeper zeta diversity decline, reflecting fewer taxa shared among sites (Fig. 6a). This pattern was consistent with lower zeta ratios in Ke ehi (Fig. 6b) and a smaller set of core families detected across all sites within the region (Fig. 6c). More taxa were shared across actively filtered samples, whereas passively collected samples showed more rapid zeta decline and lower zeta ratios (**Figure S3**). Exponential models consistently outperformed power-law models in describing zeta diversity decline, with lower AIC values observed for the exponential form in every case (ΔAIC > 15 across all markers).

Indicator species analysis identified distinct taxonomic associations across regions, sampling methods, and sites. At the regional scale, bivalve molluscs and *Porites* spp. corals were significantly associated with Ke ehi, whereas red algae including the invasive species *Acanthophora spicifera* and *Gracilaria salicornia* (Florideophyceae), were associated with Maunalua (**Table S5**; **Table S6**). Several taxonomic groups were also significantly associated with active filtration of water samples (**Table S7**). No fish or other vertebrate taxa (MFU) were identified as significant indicators at either the site or regional scale, although several culturally important and endemic species were detected (**Table S8**).

## Discussion

Our results show that eDNA metabarcoding can recover ecologically meaningful patterns in coral reef systems across markers, sampling approaches, and spatial scales. eDNA detections were consistent with visual surveys of coral cover and captured broader community structure, supporting its use for both targeted monitoring and biodiversity assessments. By embedding this technical evaluation within community-based conservation partnerships in Hawai i, this study evaluates marker performance, temporal consistency, and sampling design in applied settings, where sampling effort must reflect program goals and logistical constraints. Our findings address a broader question for marine biomonitoring: how can eDNA tools be effectively and equitably integrated into real-world conservation planning and management?

For managers focused on scleractinian coral diversity, coral-specific markers performed consistently well, detecting all genera observed in visual surveys and showing strong correspondence between eDNA read abundance and coral cover. Marker choice was less critical for presence-based monitoring but more important for tracking temporal trends. In this study, 16S showed the strongest relationship with coral cover and the most stable temporal behavior (as indicated by [17]), suggesting a reliable balance between ecological signal and variability. In contrast, ITS2 exhibited greater variability in signal intensity, which may reflect differences in amplification efficiency or sensitivity to low-abundance taxa. Temporal fluctuations in eDNA signal for individual genera across the eight timepoints were likely driven by environmental factors and detection dynamics rather than true changes in coral cover, as both visual surveys and eDNA-based community snapshots at the beginning and end of the study period were broadly consistent. Marker selection should be guided by monitoring objectives: 12S or ITS2 may offer finer taxonomic resolution in more diverse systems [24, 32], whereas 16S may be better suited for consistent temporal tracking in communities dominated by fewer genera [17]. Overall, the strong agreement between eDNA-derived and visually surveyed coral community structure supports the use of eDNA as a practical tool for reef monitoring in Hawai i, particularly in long-term programs where traditional survey methods are logistically constrained.

Baseline biodiversity surveys revealed contrasting patterns of detectability across the two additional embayments in our study. In Ke ehi, we detected a wide range of taxa, including nursery-associated fishes and endemic taxa, as well as diverse invertebrates and *limu* (algae) (**Table S8**). These detections demonstrate that even heavily urbanized reefs may retain substantial ecological function [87, 88] and potentially sustain ecologically and culturally important taxa [89–91], as these systems provide nursery, refuge, feeding, and breeding functions for nearshore species that support local fisheries [89, 92, 93]. From a management perspective, these results indicate that Ke ehi retains aspects of ecological function, supporting a diversity of native and culturally important taxa. At the same time, the coexistence of endemic species with invasive taxa such as prickly seaweed (*Acanthophora spicifera*) and gorilla ogo (*Gracilaria salicornia*) indicate a system characterized by both ecological persistence and the legacy effects of introduced species.

In Maunalua, the limited and variable detection of native fish groups, particularly large-bodied wrasses and jacks, aligns with previous visual surveys that report these taxa as rare [94]. Invasive algal species, particularly gorilla ogo and prickly seaweed, were widely detected (nine sites each), consistent with known distributions and observations from local partners [95, 96]. Despite these patterns, the relatively consistent detection of taxa across sites suggests a more homogeneous and predictable assemblage compared to Ke ehi, which may facilitate monitoring and improve the ability to detect ecological change over time. In this context, Maunalua represents a system where eDNA can be particularly effective for tracking management outcomes and documenting ecological responses to restoration efforts.

Differences in estimated taxonomic richness between these two embayments reflect variation in sampling completeness and detection frequency rather than clear differences in total diversity. After standardization by sample coverage, Maunalua was more diverse than Ke ehi; however, extrapolation indicated similar richness, except for fish and macroinvertebrates for which Maunalua had greater estimated richness. Across both embayments, zeta diversity declined exponentially with increasing site order in both embayments, indicating that shared taxa decreased rapidly across sites and that only a limited set of taxa were consistently detected region-wide. This decline is consistent with stochastic species retention and a stronger influence of local variability than a broadly shared regional assemblage [81, 83, 84]. Together, these patterns suggest that Ke‘ehi is characterized by broader but less consistently detected biodiversity, whereas Maunalua supports more consistently detected assemblages but fewer rare taxa (Fig. 6).

Passive and active sampling recovered broadly similar community structure but differed in sensitivity and consistency, demonstrating a tradeoff between logistical simplicity and analytical sensitivity [43, 44, 46]. Passive sampling captured large-scale biodiversity patterns, but exhibited greater among-sample variability, slower richness accumulation, and reduced sensitivity to rare taxa. In contrast, active filtration more consistently recovered recurrent and indicator taxa, supported by greater retention of shared taxa (**Figure S3**) and more rapid taxon accumulation per unit effort (Fig. 5). These differences may partly reflect the effects of filter clogging during active filtration, which can limit the volume of water processed and bias detection toward locally abundant taxa, whereas passive samplers integrate eDNA over time and may therefore recover a broader but more variable representation of the community (see also [97–100]). Performance of passive samplers appears to be context-dependent, shaped by interactions among sampling strategy, deployment conditions, and marker-specific detection biases [101, 102]. Although active filtration recovered higher richness in some cases, extrapolation suggests that passive sampling could achieve comparable richness with greater sampling effort. While longer deployments may increase detection, gains are not always proportional [45, 99, 103, 104]. Despite these differences, overall community structure was broadly similar between methods (Fig. 3), indicating substantial overlap in recovered assemblages.

Our findings carry several implications for the integration of eDNA into conservation practice. First, eDNA shows strong potential for coral monitoring. All three markers reliably detect Hawaiian coral genera and show strong agreement with visual estimates of coral cover, indicating broad suitability for reef monitoring. However, 16S data combined high correlation with visual surveys and stable temporal signals, demonstrating its effectiveness for single-marker biodiversity and condition monitoring in Hawai i.

Second, accessible methods expand participation in monitoring. Simple sampling approaches can broaden participation in biodiversity monitoring while still recovering comparable underlying community patterns [98, 99, 105–107], albeit with differences in the consistency and completeness of taxon detection [102, 105, 108]. In our study, passive sampling was less effective at smaller sample sizes (approx. 50 samples; Fig. 5), limiting detection of rare taxa. However, its simplicity enables greater spatial and temporal sampling effort, allowing community groups, agencies, and monitoring programs in remote regions to generate biodiversity data without specialized filtration equipment. Where resources permit, active filtration may be preferable for applications requiring higher detection consistency, particularly for rare or transient taxa, whereas passive methods may yield more variable results depending on deployment conditions [45, 101]. Accordingly, method selection should be guided by program goals and logistical constraints: passive approaches are well suited for broad-scale, highly replicated monitoring [98], whereas active filtration may be necessary for more comprehensive or fine-scale characterization.

Third, multi-marker baselines provide ecosystem context. Implementing multiple broad eDNA markers captures functionally diverse taxa (e.g., [23, 31, 33, 109]), offering a more holistic ecological perspective for management and restoration planning. However, the interpretation and application of these multi-layered datasets are most effective when grounded in local context [110]. Community partnership is therefore central to implementation. Effective applications of eDNA can emerge through collaboration with the people who know and steward reef ecosystems. Local and Indigenous ecological knowledge represent a complementary system of understanding that can inform interpretation, sampling design, and monitoring priorities [111, 112]. In this context, molecular data provide a quantitative framework that can support and extend long-standing community observations.

The adoption of eDNA monitoring remains shaped by two practical constraints: cost and validation [43, 102, 106]. In this study, baseline biodiversity surveys recovered only a fraction of estimated richness, with substantially greater sampling effort required to detect rare taxa. Although passive sampling reduced field costs and logistical barriers [100], downstream processes including DNA extraction, sequencing, and bioinformatic analysis remained resource-intensive, limiting overall sampling depth. These constraints influence how monitoring programs balance spatial coverage, temporal replication, and taxonomic completeness. In practice, eDNA is unlikely to be faster, easier, or less expensive than rapid visual assessments of coral cover. However, our results, consistent with previous visual surveys [113], show that coral assemblages change on sufficiently slow timescales that eDNA surveys conducted at multi-year intervals can provide a useful, practical, but more information-rich complement to these rapid methods. In particular, eDNA provides an independent line of evidence that can confirm broad patterns in coral cover while also capturing additional components of biodiversity not resolved in visual surveys.

Validation presents a related challenge. For corals in this study, eDNA detections were consistent with photoquadrat-based estimates of coral coverage, providing direct support for targeted monitoring [17, 114]. However, for broader community-level metabarcoding targets, including cryptic or low-abundance taxa, such one-to-one validation is often not feasible [19, 107]. In this context, validation is better viewed as convergence among multiple lines of evidence rather than strict correspondence with a single method. In this study, several detected taxa were consistent with observations from local partners, providing an additional source of corroboration. Integrating molecular data with local ecological knowledge therefore provides multiple complementary lines of evidence for ecosystem assessment, while also demonstrating the ecological and cultural dimensions of these systems. In Ke ehi, species detections provided community partners with tangible evidence of the area’s ecological value, supporting stewardship and helping to guide restoration, education, and advocacy efforts. In Maunalua, eDNA baselines may contribute to evaluating management outcomes associated with invasive algae removal, coral outplanting and Fisheries Management Area designation.

## Conclusion

Our results show that eDNA metabarcoding can recover ecologically meaningful patterns in coral reef systems while remaining flexible across monitoring goals and logistical constraints. Across markers and sampling approaches, eDNA detections were consistent with visual surveys of coral assemblages and captured broader community structure, demonstrating its use for both targeted monitoring and establishing biodiversity baselines. Beyond the technical evaluation of markers and field methods, this work demonstrates a central point for the future of eDNA in conservation: its ultimate utility is defined not in the laboratory, but in its application by the people who steward these ecosystems. For eDNA to transition from a research tool to a standard component of conservation planning, it must be evaluated not only for analytical sensitivity, but also for practicality within real-world management contexts.

This study demonstrates that eDNA can support coral reef monitoring and conservation by generating accessible, community-relevant biodiversity baselines. Co-developed with community-based organizations, these approaches are informed by local values, cultural practices, and stewardship priorities. Here, we show that eDNA can consistently track coral assemblages and generate broad biodiversity baselines in a manner accessible to local practitioners, directly supporting monitoring within the recently designated Fisheries Management Area in Maunalua Bay. These baselines provide a snapshot of reef biodiversity, equipping community partners with evidence to track restoration outcomes, inform adaptive management, and strengthen advocacy for protection in culturally significant but highly urbanized systems. In this way, eDNA can bridge molecular ecology and community-based stewardship, integrating scientific data with community and Indigenous knowledge systems to support a more complete understanding of ecosystem health. As molecular monitoring becomes more accessible, the central question is no longer whether eDNA can detect biodiversity, but how it can be implemented in ways that are accessible, scientifically robust, culturally appropriate, and operationally sustainable.

## Supplementary Material

Supplementary Files

This is a list of supplementary files associated with this preprint. Click to download.

- SupplementaryMaterial.docx
- TableS8.xlsx

## Figures and Tables

**Table 1:** Metabarcoding primers used for eDNA amplification in this study, including target community, amplicon length, gene region, study component in which each marker was applied (regional biodiversity survey and/or temporal coral monitoring), and original references.

Main target group	Primer Name	Sequence (5’→3’)	Amplicon length (bp)	Gene region	Study component	Reference
Marine invertebrates (COI)	mICOIintF	GGWACWGGWTGAACWGTWTAYCCYCC	313	Mitochondrial cytochrome c oxidase subunit I	Spatial (metazoans/macroalgae)	[55, 85]
Marine invertebrates (COI)	jgHC02198R	TANACYTCNGGRTGNCCRAARAAYCA	313	Mitochondrial cytochrome c oxidase subunit I	Spatial (metazoans/macroalgae)	[55, 85]
Eukaryotes (18S)	18S_uni_1F	GCCAGTAGTCATATGCTTGTCT	340–420	Nuclear 18S small subunit rDNA	Spatial (metazoans/macroalgae)	[57]
Eukaryotes (18S)	18S_uni_400R	GCCTGCTGCCTTCCTT	340–420	Nuclear 18S small subunit rDNA	Spatial (metazoans/macroalgae)	[57]
Corals/Sponges (16S)	HICOR16S_F1	CCGGTATGAATGGTRTCMCGA	120	Mitochondrial 16S rDNA	Spatial (metazoans/macroalgae) & Temporal (corals only)	[17]
Corals/Sponaes (16S)	HICOR16S_R1	TMCAGTAAAGYTCCATGGGG	120	Mitochondrial 16S rDNA	Spatial & Temporal	[17]
Macroalgae (23S)	p23SrV_f1	GACAGAAAGACCCTATGAA	410	Plastidial 23S rDNA	Spatial (metazoans/macroalgae)	[58]
Macroalgae (23S)	p23SrV_r1	TCAGCCTGTTATCCCTAGAG	410	Plastidial 23S rDNA	Spatial (metazoans/macroalgae)	[58]
Fish (MFU)	12S_MiFish_U_F	GTCGGTAAAACTCGTGCCAGC	163–185	Mitochondrial 12S rDNA	Spatial (metazoans/macroalgae)	[56]
Fish (MFU)	12S_MiFish_U_R	CATAGTGGGGTATCTAATCCCAGTTTG	163–185	Mitochondrial 12S rDNA	Spatial (metazoans/macroalgae)	[56]
Reef-building corals (12S)	Scle_12SF	CCAGCMGACGCGGTRANACTTA	366–465	Mitochondrial 12S rDNA	Temporal (coral only)	[32]
Reef-building corals (12S)	Scle_12SR	AAWTTGACGACGGCCATGC	366–465	Mitochondrial 12S rDNA	Temporal (coral only)	[32]
Corals/Sponges (ITS2)	SCLER5.8SForw	GARTCTTTGAACGCAAATGGC	231–437	Nuclear ITS2 rDNA	Temporal (coral only)	[86]
Corals/Sponges (ITS2)	SCLER28SRev	GCTTATTAATATGCTTAAATTCAGC	231–437	Nuclear ITS2 rDNA	Temporal (coral only)	[86]

## Data Availability

The datasets generated and/or analysed during the current study are available in the National Center for Biotechnology Information Sequence Read Archive repository (BioProject PRJNA1464528). Due to the culturally sensitive nature of sampling locations in community-managed areas of Hawai i, exact GPS coordinates have been generalized. A de-identified, aggregated dataset is available in Zenodo under DOI 10.5281/zenodo.20123379. Raw data are available from the corresponding author under a data use agreement with Mauliola Ke ehi and Mālama Maunalua.
